# Baicalein Attenuates Eosinophilic Rhinosinusitis by Suppressing ALOX15-Mediated M2 Macrophage Function

**DOI:** 10.3390/ijms27177651

**Published:** 2026-08-26

**Authors:** Lei Wang, Zhenzhen Zhu, Yuzhuo Liu, Surita Aodeng, Tianhui Kang, Weiqing Wang, Wei Lv

**Affiliations:** Department of Otolaryngology-Head and Neck Surgery, Peking Union Medical College Hospital, Chinese Academy of Medical Sciences & Peking Union Medical College, Beijing 100730, China; wanglei_199212@126.com (L.W.); entzzz@163.com (Z.Z.); lyzminminliu@163.com (Y.L.); aodengsurita@163.com (S.A.); kangth0721@126.com (T.K.)

**Keywords:** chronic rhinosinusitis, nasal polyps, baicalein, ALOX15, M2 macrophages

## Abstract

Eosinophilic chronic rhinosinusitis with nasal polyps (eCRSwNP) is characterized by type 2 inflammation, including M2 macrophage infiltration. Baicalein is a flavonoid with anti-inflammatory properties. This study aimed to investigate the therapeutic effect of baicalein in a papain-induced eosinophilic rhinosinusitis model and to determine whether it acts through arachidonate 15-lipoxygenase (ALOX15)-dependent M2 macrophage function. Clinical nasal samples from controls, non-eosinophilic CRSwNP (neCRSwNP), and eCRSwNP were analyzed for ALOX15 expression and localization. THP-1 cells were differentiated and polarized toward M2 macrophages, and the effects of baicalein on ALOX15 expression, lipid peroxidation, cytokine secretion, and transcriptomic profile were examined. The ALOX15 inhibitor PD146176 was used for target validation. A murine eosinophilic rhinosinusitis model was established by intranasal papain instillation, followed by baicalein treatment. Mucosal inflammation, IgE levels, proteoglycan 2 (PRG2)/ALOX15 expression, and immune cell infiltration were evaluated. ALOX15 was upregulated in eCRSwNP tissues and localized to CD68^+^CD206^+^ M2 macrophages. In vitro, M2 polarization increased ALOX15 expression and lipid peroxidation. Baicalein suppressed ALOX15 expression, lipid peroxidation, and the secretion of CCL22, CCL2, CXCL12, FGF-2, and IL-15. PD146176 produced similar effects, and baicalein showed no additional effect after ALOX15 blockade. RNA sequencing revealed transcriptional remodeling in M2 macrophages after baicalein treatment. In vivo, papain increased ethmoid sinus mucosal thickening, serum IgE, PRG2/ALOX15 positive cells, and infiltration of CD45^+^ immune cells, CD170^+^ eosinophils, F4/80^+^ macrophages, and B220^+^ B cells. Baicalein significantly alleviated all these pathological changes. In conclusion, baicalein attenuates papain-induced eCRSwNP-like inflammation by inhibiting lipid peroxidation and ALOX15-associated M2 macrophage secretory function. The ALOX15/M2 macrophage axis may represent a potential therapeutic target for eCRSwNP.

## 1. Introduction

Chronic rhinosinusitis (CRS) is a common inflammatory disease of the nasal cavity and paranasal sinuses. Its prevalence is estimated to be between 5.5% and 28%, imposing a heavy burden on patients’ quality of life and healthcare systems. CRS can be classified into chronic rhinosinusitis without nasal polyps (CRSsNP) and chronic rhinosinusitis with nasal polyps (CRSwNP), depending on the presence or absence of nasal polyps. In recent years, clinical focus has moved toward endotype-driven precision medicine. Eosinophilic CRSwNP (eCRSwNP) has become a unique therapeutic challenge, characterized by pronounced eosinophilic infiltration, a dominant type 2 inflammatory profile, and a high risk of recurrence after surgery [1,2,3,4].

Papain is a cysteine protease allergen that can damage the epithelial barrier and trigger eosinophilic inflammation. It is widely used to establish models of type 2 airway inflammation [5,6,7]. Tharakan et al. successfully established a mouse model of eosinophilic rhinosinusitis by intranasal papain administration. This model effectively recapitulates the hallmarks of human eCRSwNP and does not require systemic adjuvants [8].

Macrophages are highly plastic cells. Alternatively activated (M2) macrophages are closely related to type 2 inflammation, tissue repair, and fibrosis [9,10,11]. In CRSwNP, M2 macrophages promote eosinophil recruitment, B cell activation, and mucosal remodeling [12,13]. Therefore, targeting M2 macrophages may offer new therapeutic approaches for eCRSwNP.

Aberrant lipid metabolism is a driver of the functional reprogramming of inflammatory cells. Arachidonate 15-lipoxygenase (ALOX15) can catalyze the conversion of arachidonic acid and linoleic acid into various oxidized lipid mediators, participating in airway inflammation, tissue remodeling, and lipid peroxidation [14,15,16]. Our previous study confirmed that ALOX15^+^ macrophages are the key drivers of type 2 inflammation in eCRSwNP [17].

Baicalein, a major bioactive flavonoid derived from Scutellaria baicalensis, possesses anti-inflammatory, antioxidant, and immunomodulatory properties [18,19,20,21,22]. Studies have confirmed that baicalein can inhibit the expression of ALOX15 [23,24]. Based on these findings, we propose the hypothesis that baicalein may alleviate type 2 inflammation in eosinophilic rhinosinusitis by modulating ALOX15-related lipid oxidation and the function of M2 macrophages.

## 2. Results

### 2.1. ALOX15 Is Highly Expressed in eCRSwNP Tissues and Localizes to M2 Macrophages

**Figure 1:**
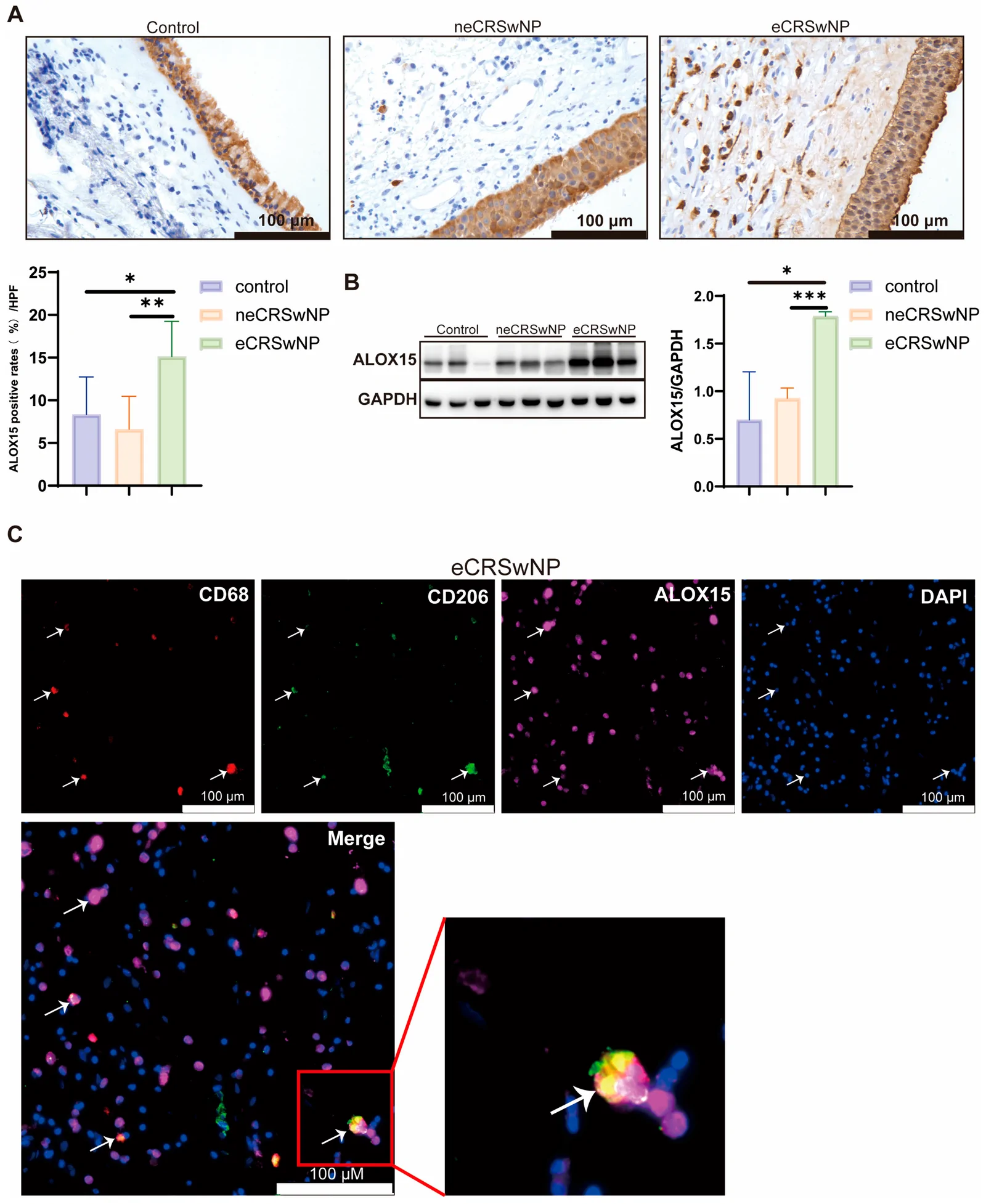
ALOX15 is highly expressed in eCRSwNP tissues and colocalizes with M2 macrophages. (**A**) Representative immunohistochemical (IHC) images showing the expression and distribution of ALOX15 in nasal mucosa tissues from the indicated groups. Scale bar = 100 μm. The bar chart at the lower left shows the quantification of positive staining area percentage (*n* = 3; * *p* < 0.05, ** *p* < 0.01). (**B**) Western blotting analysis of ALOX15 protein expression in nasal mucosa tissues from the Control, neCRSwNP, and eCRSwNP groups. The bar chart on the right shows the densitometric quantification of ALOX15 relative to GAPDH (*n* = 3; * *p* < 0.05, *** *p* < 0.001). (**C**) Immunofluorescence (IF) staining showing co-localization of M2 macrophage markers (CD68, red; CD206, green), ALOX15 (magenta), and DAPI (blue) in eCRSwNP tissues. The white arrows and the enlarged inset (red box) indicate the co-localization of ALOX15 signals with CD68^+^CD206^+^ M2 macrophages. Scale bar = 100 μm.

To characterize ALOX15 expression in eCRSwNP, we first examined clinical tissue specimens. Western blotting results showed that the ALOX15 protein level in the eCRSwNP group was significantly higher than that in the control and neCRSwNP groups (Figure 1B). IHC indicated that ALOX15 was expressed in the sinus epithelium of all groups. Notably, in the submucosal region of the eCRSwNP group, there was a specific aggregation of a large number of ALOX15-positive cells (Figure 1A). Immunofluorescence (IF) staining further confirmed that in the eCRSwNP tissue, ALOX15 was significantly colocalized with CD68^+^CD206^+^ M2 macrophages (Figure 1C and Figure S1). This indicates that M2 macrophages were an important source of ALOX15 in the submucosal layer of the eCRSwNP tissue, in line with our previous single-cell study [17] and findings from other groups [25,26,27].

### 2.2. Baicalein Suppresses ALOX15 Expression and Lipid Peroxidation in THP-1-Derived M2 Macrophages

**Figure 2:**
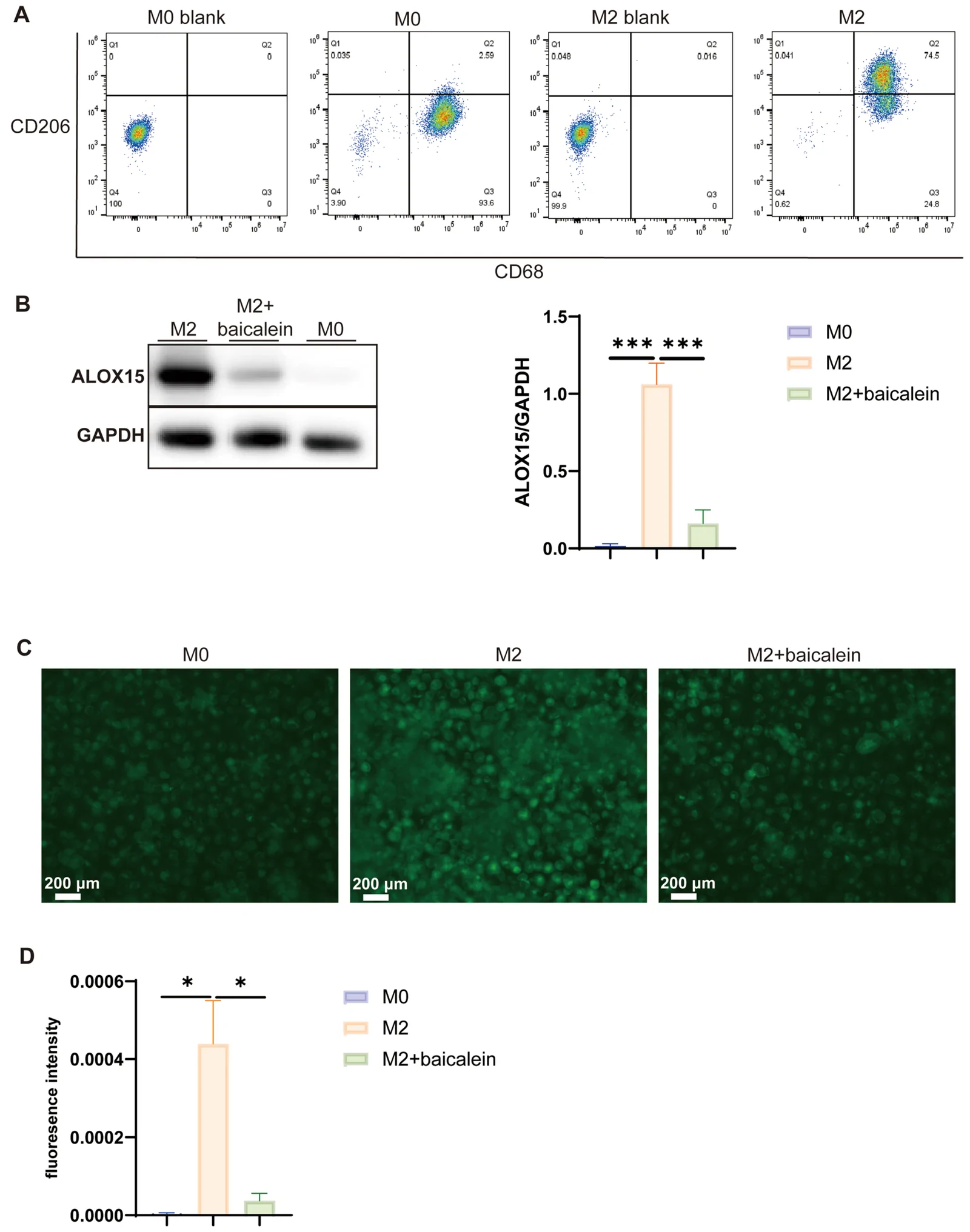
Baicalein inhibits ALOX15 expression and lipid peroxidation in THP-1-derived M2 macrophages. (**A**) Flow cytometric analysis of CD68 and CD206 expression in THP-1-derived macrophages induced toward M0 and M2 phenotypes, confirming successful M2 polarization. (**B**) Western blotting analysis of ALOX15 protein expression in M0, M2, and M2 + baicalein. The bar chart on the right shows the quantification of ALOX15/GAPDH levels (*n* = 3; *** *p* < 0.001). (**C**) Detection of intracellular lipid peroxidation levels using the BODIPY^TM^ 581/591 fluorescent probe (Green) across the M0, M2, and M2 + baicalein groups. Scale bars = 200 μm. (**D**) The bar chart below shows the quantitative analysis of fluorescence intensity (*n* = 3; * *p* < 0.05).

We established a THP-1-derived macrophage polarization model. Flow cytometry confirmed the successful M2 polarization, with a marked increase in the CD68^+^CD206^+^ cell population (Figure 2A). Western blotting showed that ALOX15 was almost undetectable in M0 macrophages. Its expression level rose sharply after M2 polarization and was significantly decreased by baicalein treatment (Figure 2B). Using the BODIPY^TM^ 581/591 C11 probe, we found that lipid peroxidation levels were higher in M2 macrophages than in M0 cells, and baicalein treatment effectively attenuated this lipid peroxidation (Figure 2C). These data indicate that M2 polarization is accompanied by enhanced ALOX15 expression and lipid peroxidation, both of which can be suppressed by baicalein.

### 2.3. Baicalein Remodels the Secretory Profile of M2 Macrophages in an ALOX15-Dependent Manner

**Figure 3:**
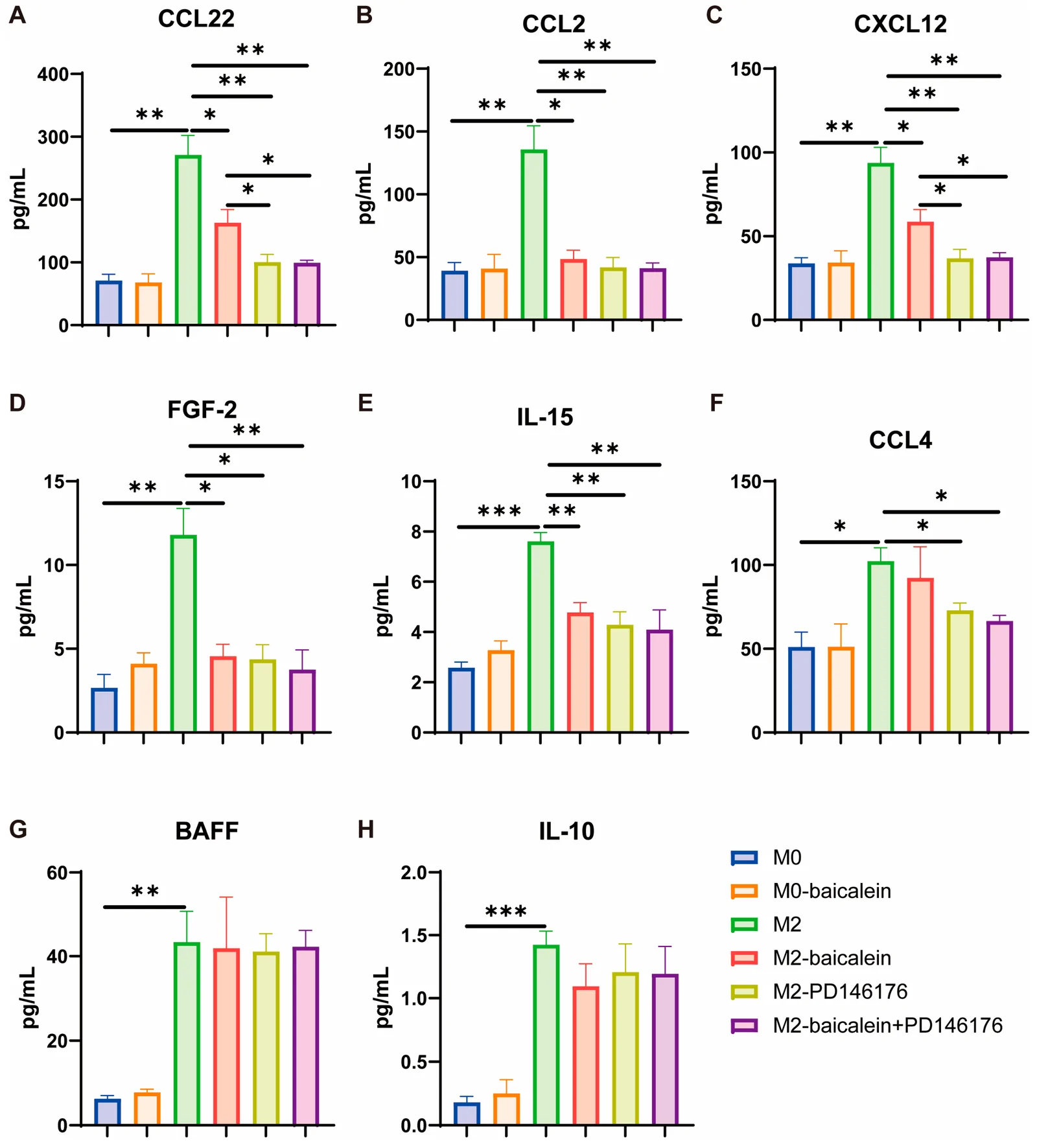
Baicalein reshapes the secretory profile of M2 macrophages in an ALOX15-dependent manner. (**A**–**H**) Luminex multiplex assay analysis of the concentrations (pg/mL) of CCL22, CCL2, CXCL12, FGF-2, IL-15, CCL4, BAFF and IL-10 in the culture supernatants of M0, M0 + baicalein, M2, M2 + baicalein, M2 + PD146176, and M2 + baicalein + PD146176 groups. (*n* = 3; * *p* < 0.05, ** *p* < 0.01, *** *p* < 0.001).

Luminex analysis revealed that the levels of CC chemokine ligand (CCL)22, CCL2, CXC chemokine ligand (CXCL)12, fibroblast growth factor 2 (FGF-2), interleukin (IL)-15, CCL4, B-cell activating factor (BAFF), and IL-10 were elevated in the culture supernatants of M2 macrophages compared with M0 cells (Figure 3A–H). Baicalein treatment significantly reduced the secretion of CCL22, CCL2, CXCL12, FGF-2, and IL-15 (Figure 3A–E), while CCL4, BAFF, and IL-10 showed a downward trend that did not reach statistical significance (Figure 3F–H). We then treated M2 macrophages with the ALOX15 inhibitor PD146176. PD146176 decreased the secretion of CCL22, CCL2, CXCL12, FGF-2, IL-15, and CCL4, producing an effect comparable to and slightly stronger than that of baicalein. Notably, when baicalein was added in the presence of PD146176, no further suppression of these factors was observed (Figure 3A–H).

In addition, M2 macrophages exhibited elevated secretion of vascular endothelial growth factor A (VEGF-A), macrophage colony-stimulating factor (M-CSF), transforming growth factor-β1 (TGF-β1), and platelet-derived growth factor-AA (PDGF-AA), whereas IL-1β and IL-6 were more abundantly secreted by M0 macrophages. In contrast, CCL24, CCL26, tumor necrosis factor-α (TNF-α), and PDGF-BB showed no significant differences between M0 and M2 macrophages. Notably, the secretion of these cytokines and growth factors was not significantly altered by baicalein or PD146176 treatment, suggesting that ALOX15-dependent regulation is selective and does not uniformly affect all M2-related secretory products (Figures S2 and S3).

### 2.4. RNA-Seq Reveals Transcriptomic Regulation of M2 Macrophages by Baicalein

**Figure 4:**
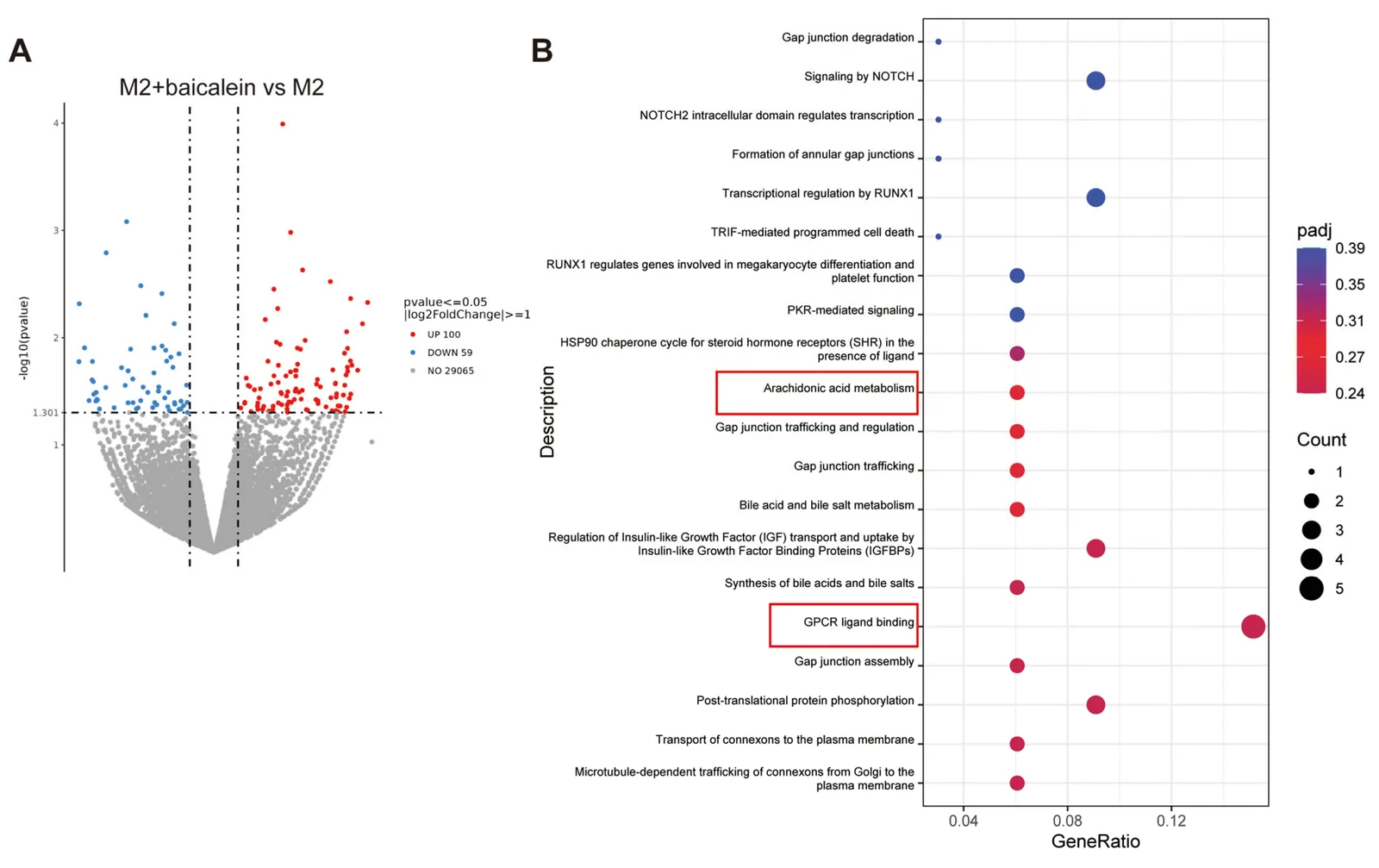
RNA-seq reveals the transcriptional regulation of M2 macrophages by baicalein. (**A**) Volcano plot of DEGs between the M2 and M2 + baicalein groups. Red dots represent upregulated genes, blue dots represent downregulated genes, and gray dots represent non-significantly changed genes. The dashed lines indicate the thresholds used for DEG screening. (**B**) Reactome pathway enrichment analysis bubble plot of the DEGs. The *x*-axis represents the GeneRatio, and the *y*-axis represents the enriched pathways.

To explore the transcriptomic impact of baicalein, we performed RNA-seq on M2 macrophages and M2 macrophages treated with baicalein. A total of 159 differentially expressed genes (DEGs) were identified, including 100 upregulated and 59 downregulated genes (Figure 4A). Reactome pathway enrichment analysis showed that these DEGs were enriched in pathways including G protein-coupled receptor (GPCR) ligand binding, arachidonic acid metabolism, gap junction-related processes, and Notch signaling (Figure 4B). The GPCR ligand binding pathway contained genes such as *CCL22*, *TAS2R5*, *UTS2R*, *CRHR2*, and *GPR68*. The arachidonic acid metabolism pathway involved *ALOX15* and *CYP8B1*. Combined with the Luminex results, these findings indicate that baicalein modulates the secretion of inflammatory factors and chemokines in M2 macrophages by regulating ALOX15.

### 2.5. Baicalein Attenuates Papain-Induced Sinus Mucosal Thickening, Goblet Cell Hyperplasia, and T Helper 2 (Th2) Cytokine Elevation in Mice

**Figure 5:**
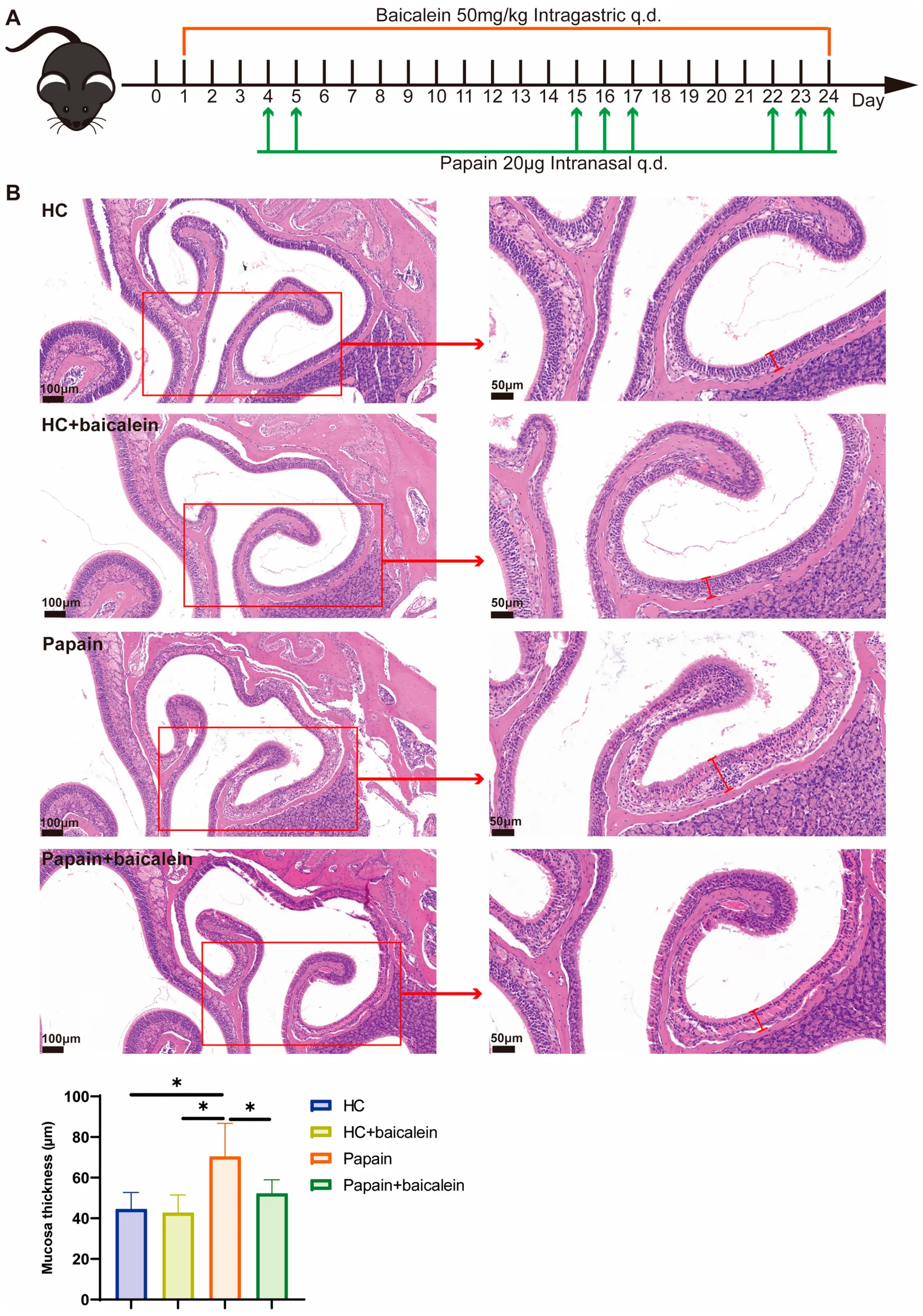
Baicalein alleviates papain-induced nasal sinus mucosal thickening in mice. (**A**) Schematic timeline of the papain-induced eosinophilic rhinosinusitis mouse model via repeated intranasal papain administration and daily intragastric baicalein intervention. (**B**) Representative H&E staining images of the ethmoid sinus mucosa from the HC, HC + baicalein, papain, and papain + baicalein groups. The red boxes indicate the magnified areas from the left panel, and the red line segments indicate mucosal thickness. Scale bars = 100 μm (**left**) and 50 μm (**right**). The bar chart below shows the quantification of mucosal thickness in the indicated groups (*n* = 5; * *p* < 0.05).

**Figure 6:**
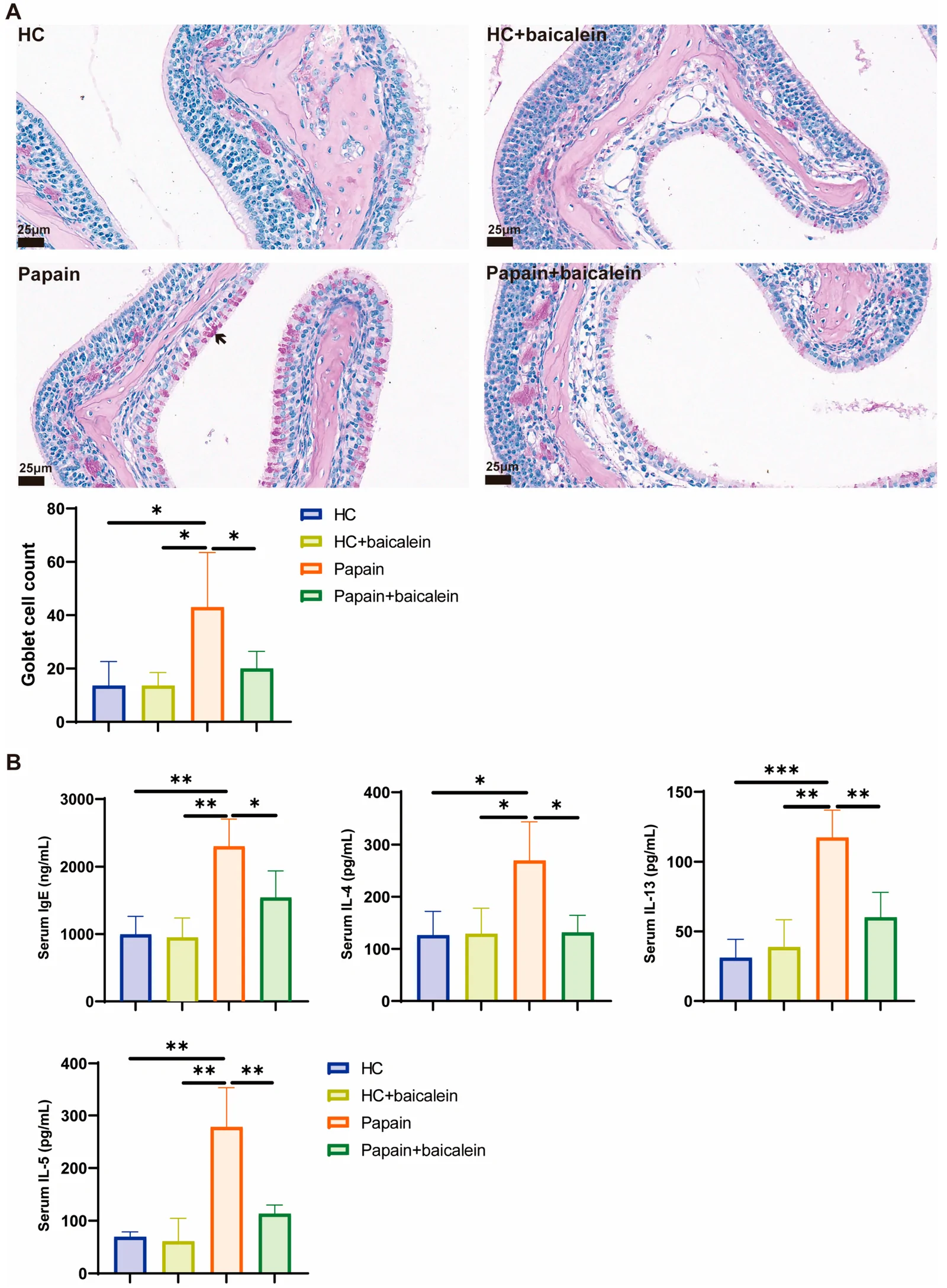
Baicalein reduces goblet cell hyperplasia and serum type 2 cytokine production in the papain-induced murine model. (**A**) Representative PAS staining images showing goblet cells in the ethmoid sinus mucosa from the HC, HC + baicalein, papain, and papain + baicalein groups. Arrows indicate PAS-positive goblet cells. Scale bar = 25 μm. The bar chart below shows the quantification of goblet cell counts (*n* = 5; * *p* < 0.05). (**B**) Serum levels of IgE, IL-4, IL-13, and IL-5 measured by ELISA across the indicated groups (*n* = 4; * *p* < 0.05, ** *p* < 0.01, *** *p* < 0.001).

We tested these effects in vivo using a mouse model of eosinophilic rhinosinusitis induced by repeated intranasal papain instillation (Figure 5A). Hematoxylin–Eosin (H&E) staining showed that the papain group displayed marked thickening of the sinonasal mucosa. Baicalein treatment substantially ameliorated this structural change (Figure 5B). To further evaluate the effect of baicalein on mucosal remodeling, we performed periodic acid–Schiff (PAS) staining to assess goblet cell hyperplasia in the sinus mucosa. The papain group exhibited a significant increase in the number of PAS-positive goblet cells compared with the healthy control (HC), indicating pronounced goblet cell metaplasia and mucus hypersecretion. Baicalein treatment markedly reduced the papain-induced goblet cell hyperplasia (Figure 6A).

We next examined whether baicalein modulates systemic Th2 cytokine responses. Serum levels of IgE, IL-4, IL-13, and IL-5 were measured by Enzyme-linked immunosorbent assay (ELISA). The papain group showed significantly elevated serum IgE levels, which were reduced by baicalein treatment. Similarly, serum IL-4, IL-13, and IL-5 levels were significantly higher in the papain group than in the HC group, and baicalein treatment significantly decreased the levels of all three Th2 cytokines (Figure 6B). Baicalein treatment alone in healthy control mice had no observable impact on sinonasal mucosal morphology, goblet cell counts, or serum IgE and Th2 cytokine levels (Figure 5 and Figure 6). These results demonstrate that baicalein effectively suppresses papain-induced type 2 immune responses, including goblet cell hyperplasia and the systemic elevation of IgE and Th2 cytokines, in the eosinophilic rhinosinusitis mouse model.

### 2.6. Baicalein Reduces Proteoglycan 2 (PRG2) and ALOX15 Expression in the Mouse Nasal Mucosa

**Figure 7:**
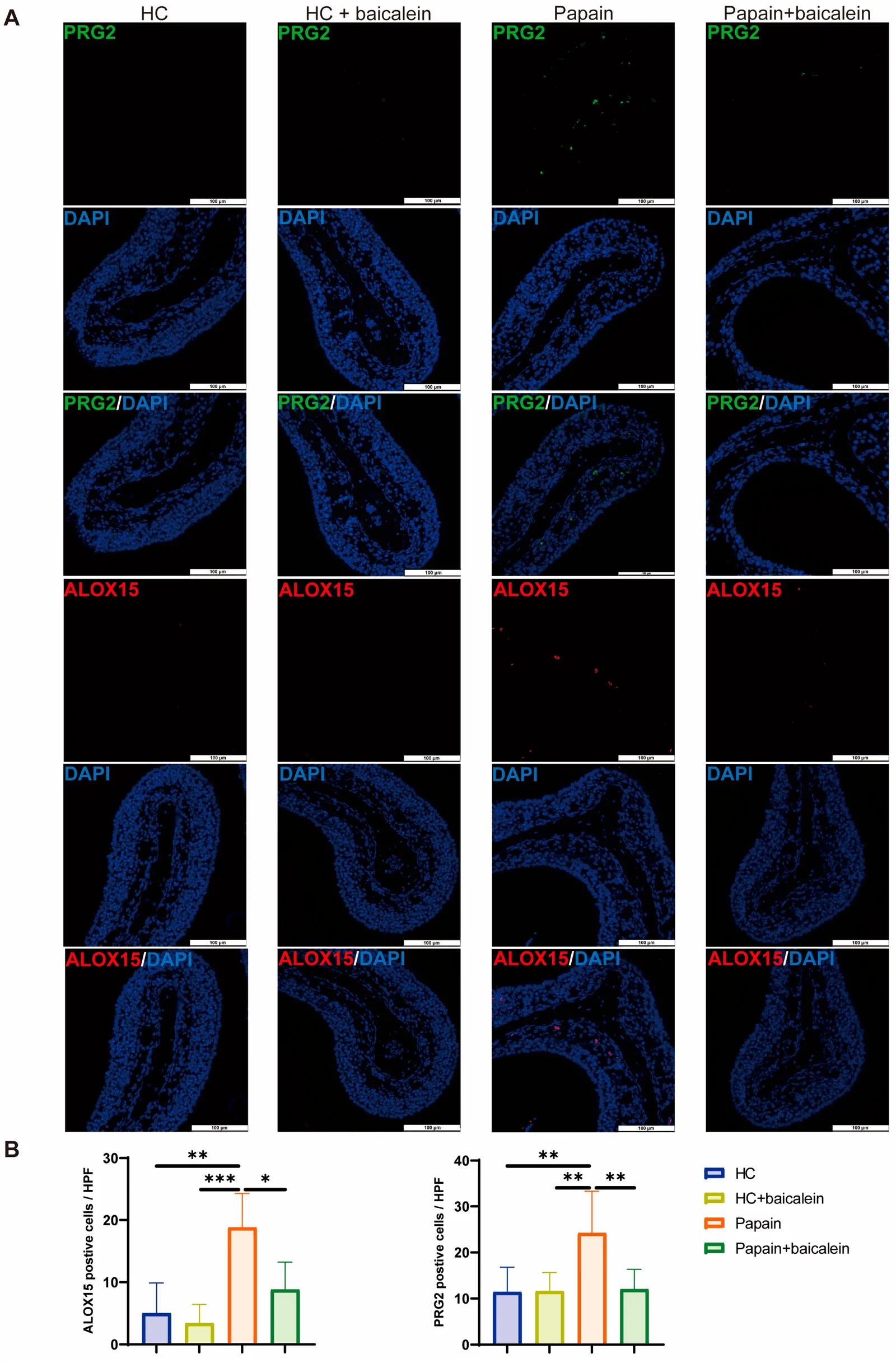
Baicalein reduces PRG2 and ALOX15 expression in mouse nasal mucosa. (**A**) IF staining for PRG2 (green) and ALOX15 (red) in the nasal mucosa. Nuclei were stained with DAPI (blue). (**B**) The quantification of PRG2 and ALOX15 positive cells per HPF (*n* = 5; * *p* < 0.05, ** *p* < 0.01, *** *p* < 0.001). Scale bar = 100 μm.

IF staining revealed weak PRG2 and ALOX15 signals in the HC group. The papain-treated group displayed a marked increase in PRG2-positive eosinophils and ALOX15-positive cells. Baicalein treatment significantly reduced the fluorescence intensity of both markers. These findings suggest that baicalein inhibits eosinophil infiltration and ALOX15 expression in this in vivo model (Figure 7).

### 2.7. Baicalein Modulates Immune Cell Infiltration in the Mouse Sinonasal Mucosa

**Figure 8:**
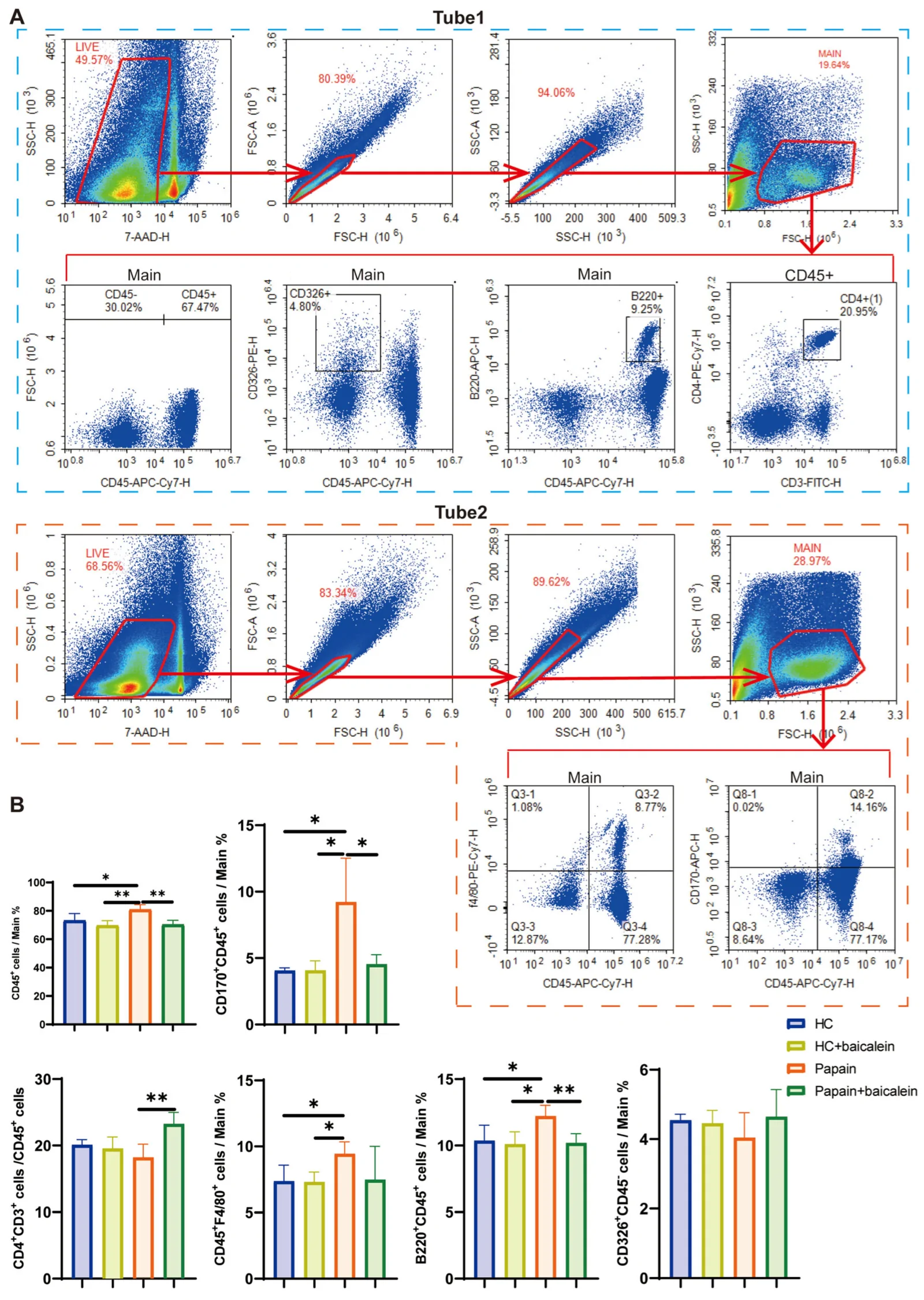
Baicalein regulates immune cell infiltration in the nasal mucosa of papain-induced mice. (**A**) Representative flow cytometry plots showing the gating strategy and immune cell subpopulations in the nasal mucosa of mice. The total leukocytes (CD45^+^) were gated, and subpopulations including eosinophils (CD170^+^), macrophages (F4/80^+^), B cells (B220^+^), CD4^+^ T cells (CD4^+^CD^3+^), and epithelial cells (CD326^+^) were further analyzed. (**B**) Quantitative bar charts showing the percentages of these specific immune cell populations in the HC, HC + baicalein, papain, and papain + baicalein groups. (*n* = 4; * *p* < 0.05, ** *p* < 0.01).

Flow cytometric analysis of sinonasal mucosa cells revealed distinct patterns among the groups. Compared with the HC group, the papain group showed significantly higher proportions of CD45^+^ total immune cells, CD170^+^CD45^+^ eosinophils, CD45^+^F4/80^+^ macrophages, and B220^+^CD45^+^ B cells. In contrast, CD4^+^CD3^+^ T cells and CD326^+^CD45^−^ epithelial cells trended downward. Baicalein treatment significantly reduced the proportions of CD45^+^ cells, eosinophils, and B cells. It also increased the proportion of CD4^+^ T cells. Both macrophages and epithelial cells showed a trend toward recovery (Figure 8A,B). These results indicate that baicalein attenuates papain-induced immune cell infiltration, with a particularly pronounced effect on eosinophil and B cell recruitment.

## 3. Discussion

The pathogenesis of eCRSwNP involves epithelial barrier disruption, type 2 immune activation, eosinophil recruitment, and tissue remodeling. When exposed to allergens, proteases, and microbial products, the nasal epithelium releases factors such as IL-25, IL-33, and thymic stromal lymphopoietin (TSLP). These factors then activate group 2 innate lymphoid cells (ILC2s), Th2 cells, and B cells, thereby promoting the production of IL-4, IL-5, IL-13, and IgE [28,29,30]. In this study, papain-challenged mice showed ethmoid mucosal thickening, elevated serum IgE levels and increased PRG2-positive cells, recapitulating the inflammatory hallmarks of eCRSwNP. Baicalein intervention significantly ameliorated these pathological changes, suggesting that it suppresses both local and systemic type 2 inflammation. Flow cytometric analysis further showed that baicalein reduced the infiltration of CD45^+^ immune cells, eosinophils, and B cells in the nasal mucosa.

ALOX15 plays crucial roles in type 2 inflammation-associated diseases [14,15,16]. IL-4 and IL-13 can upregulate ALOX15 expression in airway epithelial and immune cells [31]. In CRSwNP, ALOX15 acts as a central hub, connecting inflammation, lipid metabolism disorders, and mucosal remodeling [32,33,34]. Its expression is strikingly elevated in eCRSwNP. It also shows strong positive correlations with eosinophil counts, periostin levels, and IL-5, which makes it a valuable biomarker for CRS endotyping and recurrence prediction [32,35,36]. The pathogenic mechanisms of ALOX15 are multifaceted. It promotes eosinophil recruitment by upregulating CCL26 through the extracellular signal-regulated kinase (ERK) pathway [35,36]. It suppresses TGF-β1 via the 15(S)-HETE/peroxisome proliferator-activated receptor gamma (PPAR-γ) axis, leading to reduced collagen deposition and mucosal edema [32,33]. ALOX15^+^ M2 macrophages secrete CCL13, which induces epithelial–mesenchymal transition [25]. ALOX15 also drives ferroptosis and thus contributes to epithelial injury [34,37]. Genetic evidence further shows that loss-of-function variants in ALOX15 confer significant protection against nasal polyps [38]. The anti-IL-4Rα monoclonal antibody dupilumab can downregulate ALOX15 and ameliorate mucosal remodeling [33,39].

In CRSwNP tissues, M2 macrophages are expanded in number. However, they show impaired IL-10 secretion and excessive production of FXIII-A, which contributes to fibrin deposition and edema. They also participate in tissue remodeling by releasing matrix metalloproteinases (MMPs), VEGF-A, and other factors [40,41,42,43,44,45,46]. M2 macrophages sustain the type 2 inflammatory microenvironment through a highly active secretory network. They release CCL22 to recruit Th2 cells [47] and CCL2 to attract monocytes and additional macrophages [48]. They secrete CXCL12 to direct immune cell homing [49] and BAFF to promote B-cell survival and local IgE production [50]. Together, these factors perpetuate the chronic inflammatory loop [51,52,53,54].

We demonstrated that submucosal ALOX15-positive cells in eCRSwNP colocalized with CD68^+^CD206^+^ M2 macrophages. This identifies these macrophages as a major source of this enzyme. In vitro, M2 polarization led to a pronounced upregulation of ALOX15 and enhanced lipid peroxidation. Baicalein effectively suppressed both. The ALOX15 inhibitor PD146176 mimicked the inhibitory effect of baicalein on chemokine secretion. Combining baicalein with PD146176 produced no further suppression, suggesting that baicalein regulates M2 macrophage function primarily through the ALOX15 pathway. As a known lipoxygenase inhibitor and antioxidant, baicalein likely exerts its therapeutic effects in eosinophilic rhinosinusitis largely by targeting the ALOX15/lipid peroxidation axis in M2 macrophages.

Baicalein has been shown to modulate nuclear factor-kappa B (NF-κB), mitogen-activated protein kinase (MAPK), and signal transducer and activator of transcription (STAT) signaling pathways, thereby influencing macrophage activation [20]. In ovalbumin (OVA)-induced asthma models, it attenuates airway inflammation and mucus secretion and restores the Th1/Th2 balance [55]. It also alleviates gouty arthritis by regulating M2 macrophage polarization [56]. In our study, baicalein significantly inhibited the secretion of CCL22, CCL2, CXCL12, FGF-2, and IL-15 from M2 macrophages in an ALOX15-dependent manner. This ALOX15 dependence is further supported by the observation that the specific ALOX15 inhibitor PD146176 produced comparable inhibitory effects, and that co-administration of baicalein with PD146176 yielded no additive suppression. Although baicalein has been reported to modulate NF-κB, MAPK, and STAT pathways, these effects may be, at least in part, secondary to ALOX15 inhibition, as ALOX15-derived lipid mediators can themselves activate downstream MAPK and NF-κB cascades [57]. Nevertheless, the possibility of additional ALOX15-independent effects through direct modulation of transcription factor activity cannot be fully excluded. RNA-sequencing further revealed that baicalein regulates pathways such as GPCR ligand binding and arachidonic acid metabolism, among which *CCL22* and *ALOX15* are key regulatory nodes. These findings indicate that baicalein can reprogram M2 macrophage function and interrupt their amplification of type 2 inflammation [58].

This study has several limitations. The THP-1 cell line does not fully recapitulate the phenotype of primary nasal polyp macrophages. Future studies should validate these observations using sorted CD68^+^CD206^+^ cells from clinical specimens. Although the papain model can reproduce the key features of type 2 inflammation, it does not fully reflect the complexity of human eCRSwNP, particularly its chronic disease course and multi-factor etiology. Human polyp-derived 3D organoid cultures [59], air–liquid interface epithelial models [60], and primary nasal polyp explant cultures [61] may better recapitulate the human pathophysiology. Future studies employing a multi-model approach will be valuable for validating the therapeutic effects of baicalein across complementary experimental platforms. Additionally, the enriched pathways identified through RNA-seq analysis—including GPCR ligand binding, gap junction-related processes, and Notch signaling—were not independently validated by functional experiments such as qRT-PCR, Western blotting, or pathway-specific inhibitor studies. These targeted approaches will help to elucidate the precise molecular mechanisms underlying the transcriptomic changes.

In conclusion, this study demonstrates that baicalein ameliorates papain-induced eosinophilic rhinosinusitis by inhibiting ALOX15-associated M2 macrophage function. This attenuates lipid peroxidation, reduces the release of multiple inflammatory mediators, and diminishes eosinophil and B cell infiltration. The ALOX15/M2 macrophage axis may represent a promising therapeutic target for eCRSwNP. Baicalein has considerable potential as an anti-inflammatory strategy for eCRSwNP and warrants further development.

## 4. Materials and Methods

### 4.1. Clinical Subjects

The study enrolled patients with CRSwNP and healthy controls. The diagnosis and subtyping of CRSwNP followed the European Position Paper on Rhinosinusitis and Nasal Polyps 2020 (EPOS 2020) [1]. eCRSwNP was defined by the presence of more than 10 eosinophils per 400× high-power field in nasal polyp histopathology. Exclusion criteria included choanal polyps, fungal rhinosinusitis, allergic fungal rhinosinusitis, primary ciliary dyskinesia, and other conditions that could affect the sinonasal inflammatory milieu. Control specimens were obtained from patients without a history of rhinitis or rhinosinusitis who underwent surgery for sinus mucosal cysts, cerebrospinal fluid rhinorrhea, or benign anterior skull base tumors. This study was approved by the Ethics Committee of Peking Union Medical College Hospital (approval number: I-24PJ2606).

### 4.2. Animals

C57BL/6J mice (6–8 weeks old, half male and half female, weighing 18–22 g) were purchased from Beijing Vital River Laboratory Animal Technology Co., Ltd. (Beijing, China). The mice were housed in a specific pathogen-free (SPF) facility at Beijing Jinglai Huake Experimental Animal Center (Beijing, China; ethics approval number: JLHK-20260501-01). Mice were randomly assigned to four groups: healthy control (HC), HC + baicalein, papain, and papain + baicalein.

### 4.3. Papain-Induced Eosinophilic Rhinosinusitis Model and Baicalein Intervention

We established the mouse model based on a published protocol, with modifications [8]. Mice in the papain and papain + baicalein groups received intranasal instillations of 20 μg papain (Sigma-Aldrich, St. Louis, MO, USA; in 20 μL DPBS) on days 4, 5, 15, 16, 17, 22, 23, and 24. The HC group received an equal volume of PBS. Baicalein (Sigma-Aldrich) was prepared as a 5 mg/mL working solution. From day 1 to day 24, the papain + baicalein group was gavaged daily with baicalein at 50 mg/kg. The HC and papain groups received equivalent volumes of normal saline. On the 24th day, mice were anesthetized and then sacrificed by cervical dislocation 12 h after the final papain challenge. Blood was collected through cardiac puncture, and serum was separated for IgE measurement. Nasal mucosal tissues were obtained for subsequent experiments.

### 4.4. Histological Analysis (H&E and PAS Staining)

We collected the upper respiratory tract tissues of the mice. The tissues were fixed in 4% paraformaldehyde overnight and decalcified. Sinus mucosal tissue samples from patients were fixed using the same method. Tissues were routinely dehydrated, cleared, and embedded in paraffin. Continuous sections (5 μm) were cut, fixed on adhesive slides, and baked at 65 °C for 60 min.

For H&E staining, sections were deparaffinized in xylene and rehydrated through a series of gradient ethanols. They were stained with hematoxylin for 5 min, rinsed in tap water, differentiated in 95% ethanol, and counterstained with eosin for 2 min. The sections were then dehydrated through graded ethanols, cleared in xylene, and coverslipped with neutral gum.

The anatomical plane of coronal sectioning was standardized across all animals according to the murine sinonasal anatomy atlas [62]. All measurements were performed in the ethmoid sinus region at the same coronal level. The thickness of ethmoid sinus mucosa was measured using ImageJ software (1.53k, NIH, Bethesda, MD, USA). Mucosal thickness was defined as the distance from the apex of the epithelial cells to the lower border of the subepithelial gland zone in the lamina propria. Five independent measurements were taken per specimen by each of two investigators who were blinded to the experimental group assignments, and the final value for each animal was calculated as the mean of all measurements. For the evaluation of goblet cell hyperplasia, five ethmoid sinus mucosal sections were randomly selected per animal from PAS-stained slides, and the number of PAS-positive goblet cells was counted independently by two blinded investigators. The goblet cell count for each animal was expressed as the mean number of PAS-positive cells per section.

### 4.5. Immunohistochemistry (IHC)

We deparaffinized and rehydrated the sections. Endogenous peroxidase activity was blocked with a peroxidase blocking solution. Antigen retrieval was performed by microwave heating, and the sections were permeabilized with 0.25% Triton X-100. After serum blocking, sections were incubated overnight at 4 °C with an anti-ALOX15 primary antibody (Abcam, Cambridge, UK; ab244205, 1:2000). The next day, slides were washed with TBST and incubated with secondary antibody for 60 min at room temperature. DAB was used for color development, and nuclei were counterstained with hematoxylin. Sections were then dehydrated, cleared, coverslipped, and photographed under an upright bright-field Leica DM6 B microscope (Leica Microsystems, Wetzlar, Germany). The percentage of IHC-positive staining area relative to the total tissue area was quantified using ImageJ software.

### 4.6. Immunofluorescence (IF)

We used an IF procedure similar to IHC, but without the peroxidase blocking step. The primary antibodies were against PRG2 (Invitrogen, Thermo Fisher Scientific, Waltham, MA, USA; PA5-102628, 1:500), ALOX15 (Abcam, ab244205, 1:1000), CD68 (Thermo Fisher Scientific, MA5-12407, 1:200) and CD206 (R&D Systems, Inc., Minneapolis, MN, USA; MAB2534, 1:200). The secondary antibodies were Alexa Fluor 488 (Invitrogen, A-11008, 1:2000), Alexa Fluor 555 (Invitrogen, A-21422, 1:2000) and Alexa Fluor 647 (Cell Signaling Technology, Inc., Danvers, MA, USA; #4418, 1:2000). Nuclei were counterstained with DAPI. Images were acquired using a Leica DMi8 (Leica Microsystems) inverted fluorescence microscope.

### 4.7. Preparation of Single-Cell Suspension from Mouse Nasal Mucosa

Nasal mucosal tissues were minced on ice into pieces smaller than 1 mm^3^ and transferred to DMEM digestion medium containing 3 mg/mL collagenase I, 0.2 mg/mL DNase I, 0.5 mg/mL hyaluronidase, and 2 mg/mL dispase. Digestion was performed at 37 °C with shaking at 170 rpm for 20 min. The cell suspension was filtered through a 40 μm cell strainer and centrifuged. The pellet was incubated with red blood cell lysis buffer on ice for 3 min. Cells were washed with PBS and counted.

### 4.8. Flow Cytometry of Mouse Nasal Mucosa

Single-cell suspensions were stained with fluorochrome-conjugated antibodies: CD3-FITC (100203), CD4-PE/Cy7 (100527), CD45-APC/Cy7 (103116), CD326/EpCAM-PE (118205), F4/80-PE/Cy7 (123114), B220-APC (103212), and CD170/Siglec-F-APC (155507), all from BioLegend (San Diego, CA, USA). Data were acquired on a NovoCyte 3005 flow cytometer (Agilent Technologies, San Diego, CA, USA) and analyzed with FlowJo software (v10.8.1, BD Biosciences, Ashland, OR, USA). Our gating strategy was as follows: after excluding debris and dead cells, single cells were gated. We then analyzed the proportions of CD45^+^ total immune cells, CD170^+^ eosinophils, F4/80^+^ macrophages, B220^+^ B cells, CD3^+^CD4^+^ T cells, and CD326^+^ epithelial cells.

### 4.9. Enzyme-Linked Immunosorbent Assay (ELISA)

Serum IgE levels were measured using a Mouse IgE ELISA Kit (Sangon Biotech, Shanghai, China; D721207) according to the manufacturer’s instructions. For the measurement of serum Th2 cytokines in the eosinophilic rhinosinusitis mouse model, Mouse IL-4 (ml064310), IL-5 (ml063157), and IL-13 (ml106729) ELISA Kits (Shanghai MLBIO Biotechnology Co., Ltd., Shanghai, China) were used. The concentrations of TGF-β1, PDGF-AA, and PDGF-BB in the cell culture supernatants of THP-1-derived macrophages were measured using Human TGF-β1 (ml022522), PDGF-AA (ml106314), and PDGF-BB (ml105299) ELISA Kits (Shanghai MLBIO Biotechnology Co., Ltd.). All procedures were performed according to the manufacturers’ instructions. Absorbance was read with a microplate reader, and concentrations were calculated from standard curves.

### 4.10. THP-1-Derived Macrophage Culture and M2 Polarization

The THP-1 monocyte cell line (National Bio-Medical Cell-Line Resource Center, Beijing, China) was cultured in RPMI-1640 medium supplemented with 10% fetal bovine serum (FBS) at 37 °C with 5% CO_2_. For macrophage differentiation, THP-1 cells were treated with 100 nM phorbol-12-myristate-13-acetate (PMA; Sigma-Aldrich). M2 polarization was then induced by stimulation with IL-4 (20 ng/mL; PeproTech, Rocky Hill, NJ, USA) and IL-13 (20 ng/mL; PeproTech). For baicalein treatment, 20 μM baicalein was added after M2 polarization, and cells were cultured for an additional 24 h; the control group received an equal volume of DMSO. To verify the role of ALOX15 in the effects of baicalein, an additional group was treated with the ALOX15 inhibitor PD146176 (10 μM; Sigma-Aldrich), either alone or in combination with baicalein.

### 4.11. Flow Cytometry of THP-1 Cells

Cells were collected and resuspended in PBS at 1 × 10^6^ cells/mL. After blocking with 1% bovine serum albumin (BSA), cells were incubated with PE anti-human CD206 (BioLegend; 321106) and APC anti-human CD68 (BioLegend; 333810) at 4 °C in the dark for 30 min. Following a washing step, samples were analyzed on a flow cytometer, and data were processed with FlowJo software.

### 4.12. Lipid Peroxidation Assay

Cells were stained with 10 μM BODIPY^TM^ 581/591 C11 probe (Thermo Fisher Scientific) at 37 °C for 30 min, washed with PBS, and imaged using an EVOS microscopy system (Thermo Fisher Scientific) under the 488 nm channel. Fluorescence intensity was quantified with ImageJ software.

### 4.13. Western Blotting

Total protein was extracted from tissues or cells on ice using RIPA lysis buffer. Denatured proteins were separated by SDS-PAGE (75 V for stacking gel, 120 V for separating gel) and transferred onto nitrocellulose membranes by wet transfer (220 mA, 2.5 h). We blocked the membranes with 5% non-fat milk and probed them with primary antibodies: anti-ALOX15 (Abcam; 1:2000) and anti-GAPDH (Santa Cruz Biotechnology, Dallas, TX, USA; sc-137179, 1:2000). We then incubated the membranes with HRP-conjugated secondary antibodies. Signals were developed with ECL reagent. Band intensities were analyzed using ImageJ software.

### 4.14. RNA Sequencing (RNA-Seq)

M2 macrophages treated with baicalein or DMSO for 24 h were collected in TRIzol reagent (Thermo Fisher Scientific) (three biological replicates per group). RNA-seq was performed by Novogene Co., Ltd. (Beijing, China). After quality control, read alignment, and quantification, DEGs were identified using the criteria |log_2_FoldChange| ≥ 1 and *p* ≤ 0.05. Reactome pathway enrichment analysis was carried out with the clusterProfiler package. The RNA-seq data will be deposited in a public database upon publication, and the accession number will be provided in the final manuscript.

### 4.15. Luminex

Cell culture supernatants were assayed using a 65-plex Human Cytokine/Chemokine Panel (Thermo Fisher Scientific) according to the manufacturer’s instructions. Data were acquired on a Luminex 200^TM^ system (Luminex Corporation, Austin, TX, USA), and concentrations were calculated using a 5-parameter logistic curve.

### 4.16. Statistical Analysis

All statistical analyses were performed using GraphPad Prism 9 software (GraphPad Software, San Diego, CA, USA). Data are expressed as mean ± SD. For two-group comparisons, Student’s *t*-test was applied to normally distributed data, while the Mann–Whitney U test was used for non-parametric data. A *p* value < 0.05 was considered statistically significant. Significance levels are denoted as follows: * *p* < 0.05, ** *p* < 0.01, *** *p* < 0.001, **** *p* < 0.0001.

## Data Availability

The original contributions presented in this study are included in the article/Supplementary Materials. Further inquiries can be directed to the corresponding author.

## Supplementary Materials

The following supporting information can be downloaded at: https://www.mdpi.com/article/10.3390/ijms27177651/s1.

## Abbreviations

The following abbreviations are used in this manuscript:

ALOX15	arachidonate 15-lipoxygenase
BAFF	B-cell activating factor
CCL	CC chemokine ligand
CRS	chronic rhinosinusitis
CRSwNP	chronic rhinosinusitis with nasal polyps
CXCL	CXC chemokine ligand
DEGs	differentially expressed genes
eCRSwNP	eosinophilic chronic rhinosinusitis with nasal polyps
ELISA	Enzyme-linked immunosorbent assay
FGF-2	fibroblast growth factor 2
GPCR	G protein-coupled receptor
HC	healthy control
H&E	hematoxylin and eosin
IF	immunofluorescence
IHC	immunohistochemistry
IL	interleukin
MAPK	mitogen-activated protein kinase
neCRSwNP	non-eosinophilic chronic rhinosinusitis with nasal polyps
NF-κB	nuclear factor-kappa B
PDGF	platelet-derived growth factor
PRG2	proteoglycan 2
PAS	periodic acid–Schiff
RNA-seq	RNA sequencing
STAT	signal transducer and activator of transcription
TGF-β1	transforming growth factor beta 1
Th2	T helper 2
TSLP	thymic stromal lymphopoietin
VEGF-A	vascular endothelial growth factor A
