# Supplementary material for: Baicalein Attenuates Eosinophilic Rhinosinusitis by Suppressing ALOX15-Mediated M2 Macrophage Function
Source: Int J Mol Sci. 2026 Aug 26;27(17):7651. doi: 10.3390/ijms27177651 (PMC13566850; doi:10.3390/ijms27177651)
Supplement: Supplementary file 1 [file ijms-27-07651-s001.zip › Supplementary Figure Legends.pdf]

Figure S1. Immunofluorescence staining of ALOX15 and M2 macrophage markers in Control and neCRSwNP tissues. (A–B) Representative immunofluorescence (IF) images showing the localization of M2 macrophage markers (CD68, red; CD206, green), ALOX15 (magenta), and DAPI (blue) in nasal mucosa tissues from the Control (A) and neCRSwNP (B) groups. Scale bar = 100 μm. In contrast to the eCRSwNP tissues (shown in Figure 1C)

Figure S2. Luminex multiplex analysis of cytokine and chemokine secretion in THP-1-derived macrophages. THP-1 cells were differentiated into M0 macrophages and polarized to M2 macrophages as described in Figure 3. The concentrations of (A) VEGF-A, (B) M-CSF, (C) CCL24, (D) CCL26, (E) IL-1β, (F) IL-6, and (G) TNF-α in the cell culture supernatants were measured using Luminex multiplex immunoassay. (n=3; *P < 0.05).

Figure S3. Effects of baicalein and PD146176 on the secretion of TGF-β1, PDGF-AA, and PDGF-BB in THP-1-derived macrophages. THP-1 cells were differentiated into M0 macrophages and polarized to M2 macrophages as described in Figure 3. The concentrations of (A) TGF-β1, (B) PDGF-AA, and (C) PDGF-BB in the cell culture supernatants were measured by ELISA. (n=3; *P < 0.05, ***P < 0.001).
